# Publisher Correction: Following excited-state chemical shifts in molecular ultrafast x-ray photoelectron spectroscopy

**DOI:** 10.1038/s41467-022-28584-2

**Published:** 2022-03-09

**Authors:** D. Mayer, F. Lever, D. Picconi, J. Metje, S. Alisauskas, F. Calegari, S. Düsterer, C. Ehlert, R. Feifel, M. Niebuhr, B. Manschwetus, M. Kuhlmann, T. Mazza, M. S. Robinson, R. J. Squibb, A. Trabattoni, M. Wallner, P. Saalfrank, T. J. A. Wolf, M. Gühr

**Affiliations:** 1 Institut für Physik und Astronomie, Universität Potsdam, 14476 Potsdam, Germany; 2 Institut für Chemie, Universität Potsdam, 14476 Potsdam, Germany; 3 Deutsches Elektronen Synchrotron (DESY), 22607 Hamburg, Germany; 4 Center for Free-Electron Laser Science (CFEL), Deutsches Elektronen Synchrotron (DESY), 22607 Hamburg, Germany; 5 The Hamburg Centre for Ultrafast Imaging, Universität Hamburg, 22761 Hamburg, Germany; 6 Institut für Experimentalphysik, Universität Hamburg, 22761 Hamburg, Germany; 7 Heidelberg Institute for Theoretical Studies, HITS gGmbH, 69118 Heidelberg, Germany; 8 Department of Physics, University of Gothenburg, SE-41296 Gothenburg, Sweden; 9 European XFEL, 22869 Schenefeld, Germany; 10 Stanford PULSE Institute, SLAC National Accelerator Laboratory, Menlo Park, CA 94025 USA

**Keywords:** Atomic and molecular interactions with photons, Chemical physics, Chemical physics

Correction to: *Nature Communications* 10.1038/s41467-021-27908-y, published online 11 January 2022.

The original PDF version of this Article contained errors in Figure 2 and Figure 3 due to an error in the publication process.

In the original version of Figure 2, the difference signal values in panel b were reported incorrectly.

The correct version of Figure 2 is:
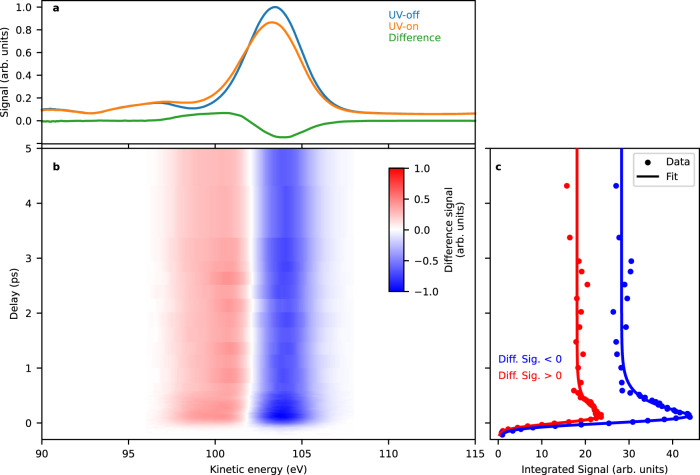


Which replaces the previous incorrect version:
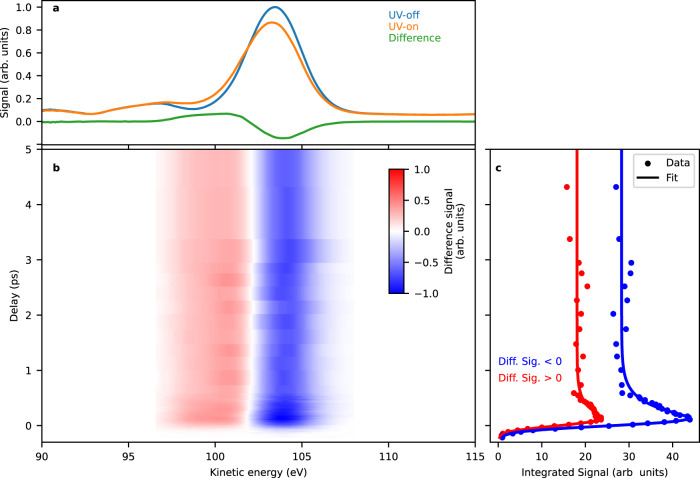


In the original version of Figure 3, the difference signal values in panel a were reported incorrectly.

The correct version of Figure 3 is:
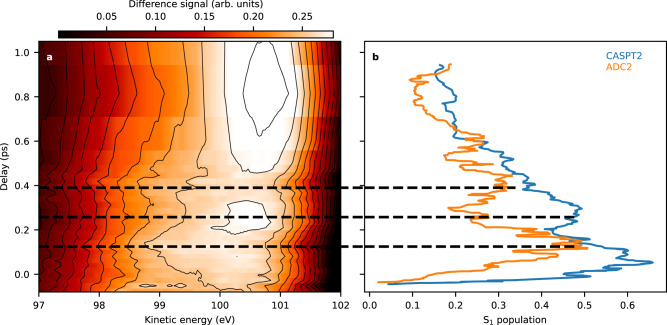


Which replaces the previous incorrect version:
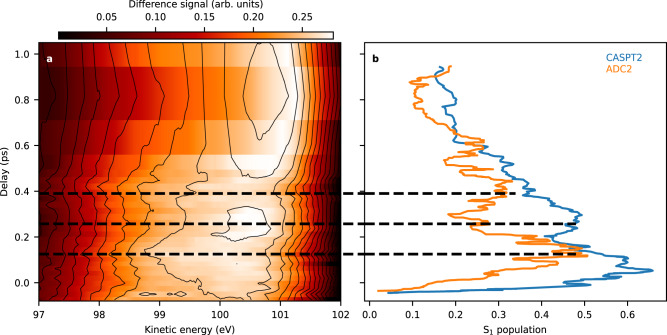


This has been corrected in the PDF version of the Article.

