# Supplementary material for: Following excited-state chemical shifts in molecular ultrafast x-ray photoelectron spectroscopy
Source: Nat Commun. 2022 Jan 11;13:198. doi: 10.1038/s41467-021-27908-y (PMC8752854; doi:10.1038/s41467-021-27908-y)
Supplement: Supplementary file 1 — Supplementary information [file 41467_2021_27908_MOESM1_ESM.pdf]

Supplementary information

Following excited-state chemical shifts in molecular ultrafast x-ray photoelectron spectroscopy

D. Mayer[1+], F. Lever[1+], D. Picconi[2,*] , J. Metje[1], S. Alisauskas[3], F. Calegari[4,5,6], S. Düsterer[3], C. Ehlert[7], R. Feifel[8], M. Niebuhr[1], B. Manschwetus[3] , M. Kuhlmann[3], T. Mazza[9], M. S. Robinson[1,4,5], R. J. Squibb[8], A. Trabattoni[4] , M. Wallner[8], P. Saalfrank[2], T. J. A. Wolf[10] and M. Gühr[1,*]

1 Institut für Physik und Astronomie, Universität Potsdam, 14476 Potsdam, Germany
2 Institut für Chemie, Universität Potsdam, 14476 Potsdam, Germany
3 Deutsches Elektronen Synchrotron (DESY), 22607 Hamburg, Germany
4 Center for Free-Electron Laser Science (CFEL), Deutsches Elektronen Synchrotron (DESY), 22607 Hamburg, Germany
5 The Hamburg Centre for Ultrafast Imaging, Universität Hamburg, 22761 Hamburg, Germany
6 Institut für Experimentalphysik, Universität Hamburg, 22761 Hamburg, Germany
7 Heidelberg Institute for Theoretical Studies, HITS gGmbH, 69118 Heidelberg, Germany
8 Department of Physics, University of Gothenburg, SE-41296 Gothenburg, Sweden
9 European XFEL, 22869 Schenefeld, Germany
10 Stanford PULSE Institute, SLAC National Accelerator Laboratory, 94025 Menlo Park, United States of America

+ Contributed equally
** correspondence should be addressed to david.picconi@uni-potsdam.de and mguehr@uni-potsdam.de*

Contents

Supplementary Discussion 1: Fits of the delay-dependent signals 2

Supplementary Discussion 2: Magnetic bottle sensitivity 4

Supplementary Discussion 3: Spectral oscillations 6

Supplementary Discussion 4: Quantum chemical results 7

Supplementary Discussion 5: Calculated state- and geometry-dependent pump-probe XPS spectra 13

Supplementary Discussion 6: Potential model fits 15

Supplementary Discussion 7: Exceptions in the clustering according to electronic state 17

Supplementary Discussion 8: Pulse energy scans 18

Supplementary Discussion 9: Data handling 19

Supplementary References 20

Supplementary Discussion 1: Fits of the delay-dependent signals

The integral of the difference signal and the amplitudes from the double Gaussian fit show similar delay-dependent behaviour and have thus been fitted with the same function:

$$S(t) = \sum_{i=1}^{2} A_i \cdot exp\left\{-\tau_i^{-1} \cdot (t - t_0)\right\} exp\left\{0.5 \cdot \tau_i^{-2} \cdot \sigma^2\right\} \cdot \left[1 + erf\left(\frac{t - t_0 - \tau_i^{-1} \cdot \sigma^2}{\sqrt{2}\sigma}\right)\right] \quad (1)$$

The equation represents a Gaussian time-uncertainty function convoluted with two exponential decays. Here, A_i and τ_i are the amplitude and decay constant of the i-th component. t_0 is the time-zero i.e., the overlap between pump and probe pulse, and σ describes the temporal resolution (with σ^2 being the variance of the Gaussian function). The erf is the Gauss error function. The fitting range was restricted to -0.2 to +10 ps.

First, the integral of the absolute difference signal ($S_{\mathrm{int}}^{\mathrm{abs}}$) was fitted. The fitted t_0 value was used to correct the delays. Then, the integral of the positive (S_{int}^{+}) and negative (S_{int}^{-}) contributions to the difference spectra were fitted with a fixed time uncertainty using the value from $S_{\mathrm{int}}^{\mathrm{abs}}$. The results of all five fits are summarised in Supplementary Table 1.

The full width at half maximum of the Gaussian time-uncertainty function is 190 ± 10 fs. This value includes the pulse duration of the UV pump and the x-ray probe pulse as well as temporal jitter [1,2]. The time constant of the first decay component τ_1 lies between 200 and 300 fs for all fits with an average value of 235 ± 10 fs. For the second decay constant τ_2, only two fits give values within the picosecond range (~200 ps). The other values are within nano- or even microsecond range with similarly large fitting errors (noted with >1000 in table S1). Increasing the delay range for the fit does not improve the values for τ_2. The values for τ_1 stay similar. A normalized plot in Supplementary Figure 1, allowing for a better comparison of the positive and negative signal decay, confirms that the observed decays on the positive and negative lobe are equal.

	S_{int}^{abs}	S_{int}^{-}	S_{int}^{+}
σ / fs	81± 5	-	-
t_0 / fs	-56 ± 4	2.0 ± 4.0*	-6 ± 6*
A_1	43 ± 4	31 ± 2	12 ± 2
τ_1 / fs	225 ± 30	250 ± 20	220 ± 40
A_2	46.5 ± 2	28.2 ± 0.8	17.5 ± 0.6
τ_2 / ps	200 ± 140	>1000	>1000
offset	1.8 ± 1.0	1.0 ± 0.6	0.70 ± 0.6

Supplementary Table 1. Fitted parameters of delay dependent amplitudes and integrals. “-” indicates that the value from S_{int}^{abs} has been used. t_0 values with an asterisk (*) are evaluated after correcting the delays with t_0 value from S_{int}^{abs}.

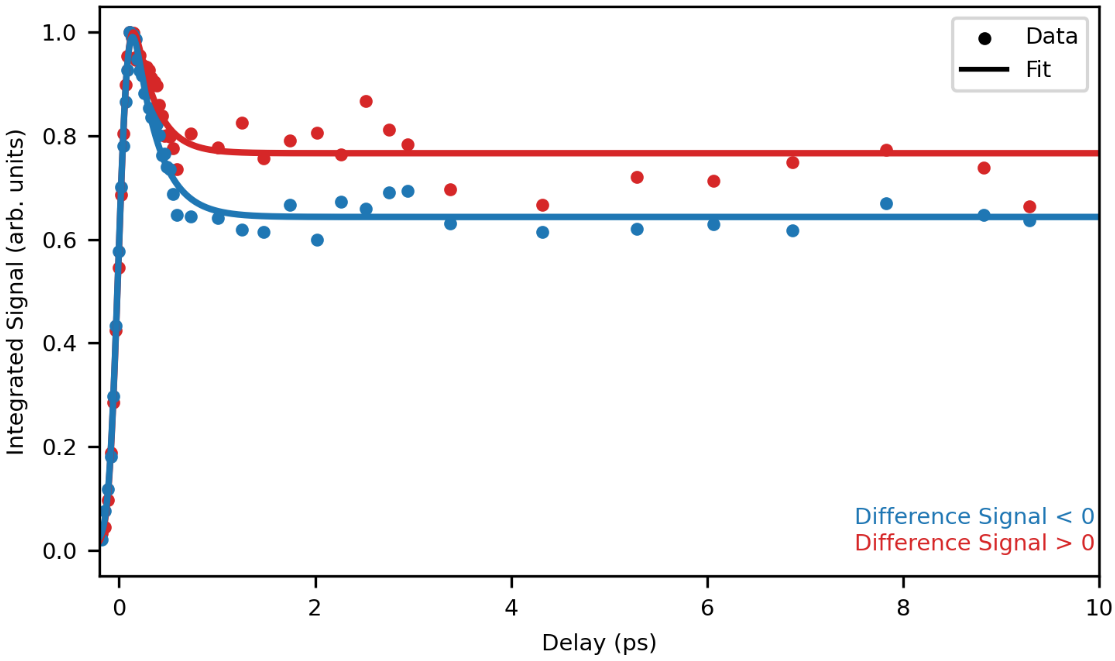

Supplementary Figure 1. Integrated (absolute) signal for positive (red) and negative lobe (blue) of the S 2*p* difference spectrum normalized on the maximum.

Supplementary Discussion 2: Magnetic bottle sensitivity

The data shown in the following plots (Suppl. Fig. 2 to 4) were recorded on a follow up beamtime to investigate the influence of different experimental parameters on the photoelectron spectra.

In Suppl. Fig. 2, the solenoid (coil) current and thus the magnetic field inside the flight tube was changed. The electron spectra were recorded with a photon energy of 272 eV at a delay of 200 ps and a retardation voltage of -80 V. Panel a) shows the normalised difference spectra of the sulphur 2*p* photoline. The positive (red) and negative (blue) contributions broaden with increasing magnetic field and also shift towards lower kinetic energy. Additionally, panel b) shows the integrals of the positive and negative contributions of the spectra. The two lobes change signal strength in a counter-oscillatory way. Especially the negative lobe is stronger than the positive one at 200 mA which was the value also used for the data presented in this paper.

In Suppl. Fig. 3, the coil current was kept constant at 200 mA and instead the retardation voltage was scanned across -80 V, while the kinetic energy is corrected for the change in retardation potential. Again, panel a) shows the difference spectra and b) the integrals of the positive and negative contributions. The counter-oscillation of the two lobe integrals is again observed. This is also the case for a photon energy scan around 272 eV shown in Suppl. Fig. 4 where the coil current and retardation voltage are kept constant at values of 200 mA and -80 V, respectively.

Our systematic investigations of the difference spectra for various experimental settings exhibit the influence of cyclotron resonances on the relative amplitudes in the MBES, an aspect which is well known for this kind of electron spectrometer[1]. In future runs, the characterisation might be used to calibrate the MBES sensitivity.

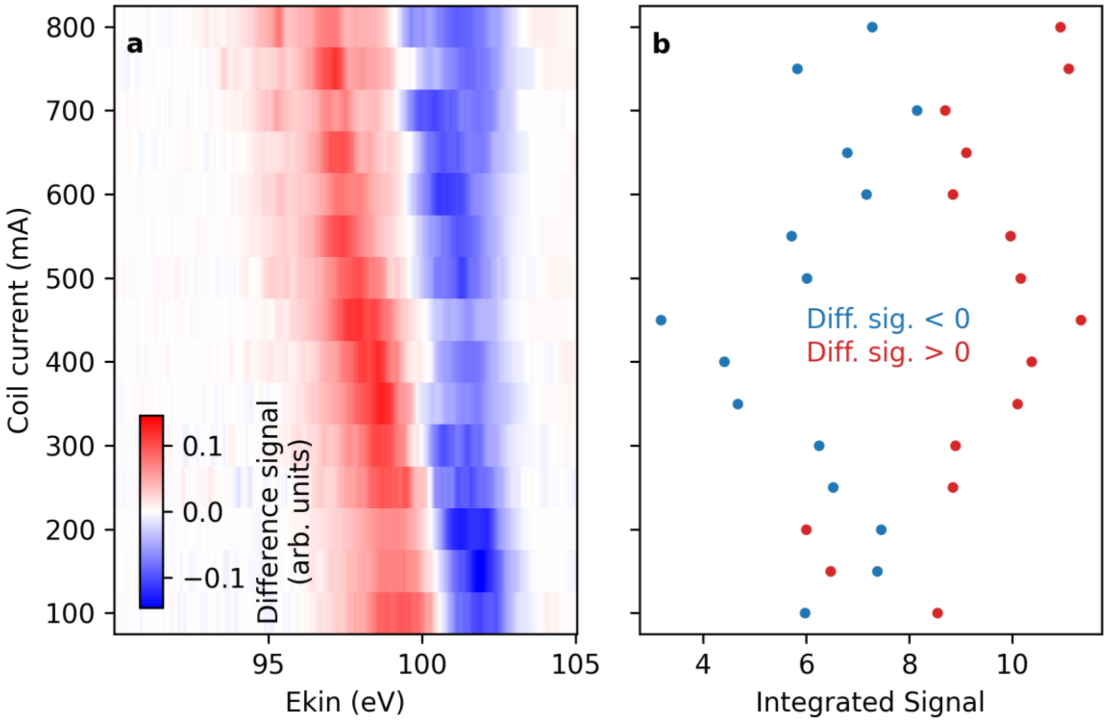

Supplementary Figure 2. Scan of the coil current on the flight tube at 272 eV x-ray photon energy and -80 V retardation. a: false-color representation of the difference spectra of the photoline. b: integrated signal of positive (red) and negative (blue) contributions.

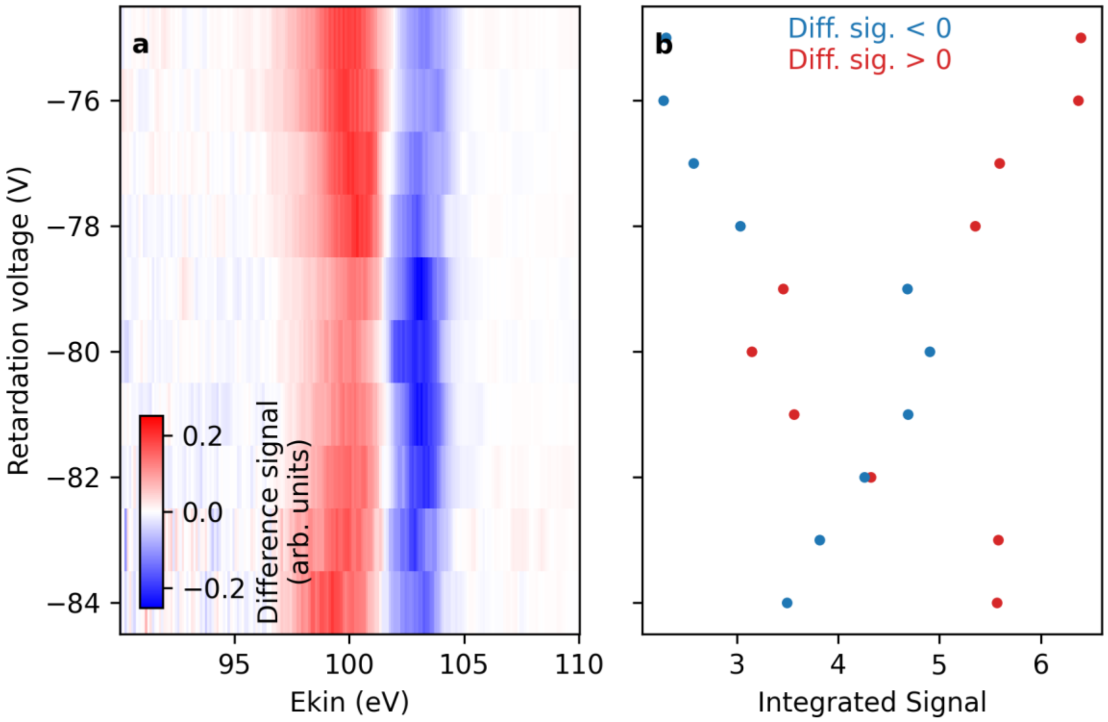

Supplementary Figure 3. Scan of the retardation voltage at 272 eV and 200 mA coil current. a: false-color representation of the difference spectra of the photoline. b: integrated signal of positive (red) and negative (blue) contributions.

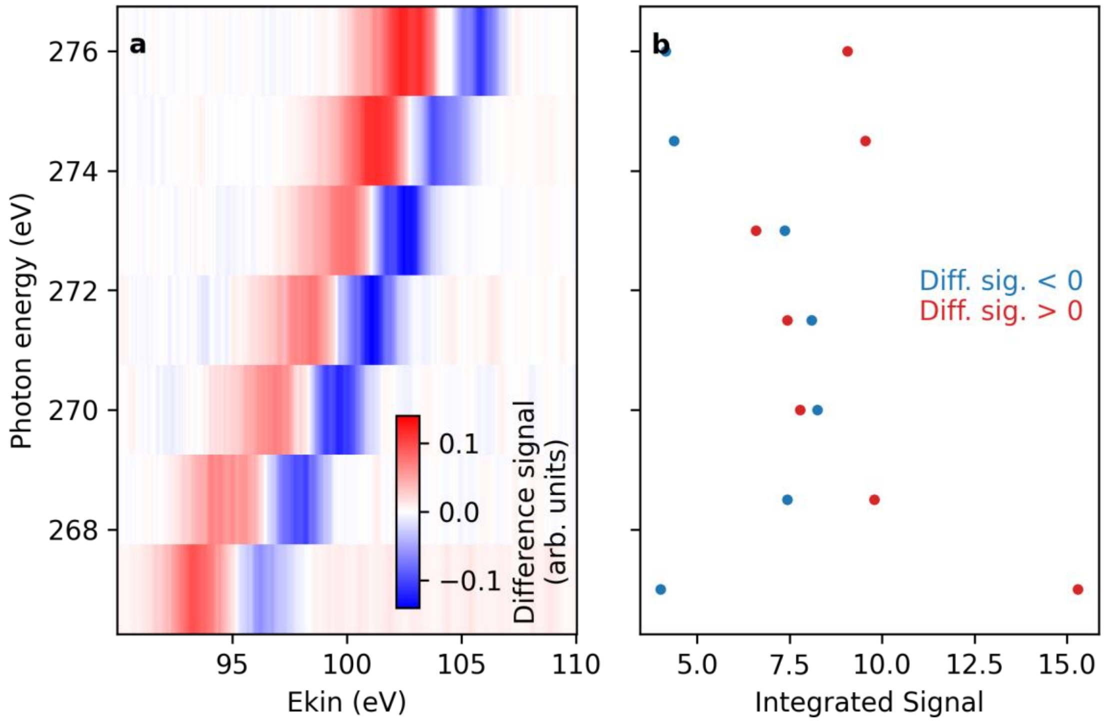

Supplementary Figure 4. Scan of the photon energy at 80V retardation and 200mA coil current. a: false-color representation of the difference spectra of the photoline. b: integrated signal of positive (red) and negative (blue) contributions.

Supplementary Discussion 3: Spectral oscillations

To reduce the complexity of the data shown in Fig. 3, we show lineouts at the positive lobe's low and high kinetic energy edges at 98.8 eV (blue) and 101.2 eV (orange) in Suppl. Fig. 5. In the delay region described above, both lineouts show an oscillation. They occur with opposite phase as expected for spectral shifts. At 150 and 400 fs, the underlying photoelectron signal shifts to lower kinetic energies, increasing the lineout at 98.8 eV while decreasing the lineout at 101.2 eV. In between these times, the photoelectron signal shifts to higher kinetic energies, reducing the lineout at 98.8 eV and increasing the 101.2 eV lineout. At higher delays, the step size is too coarse to resolve such oscillation.

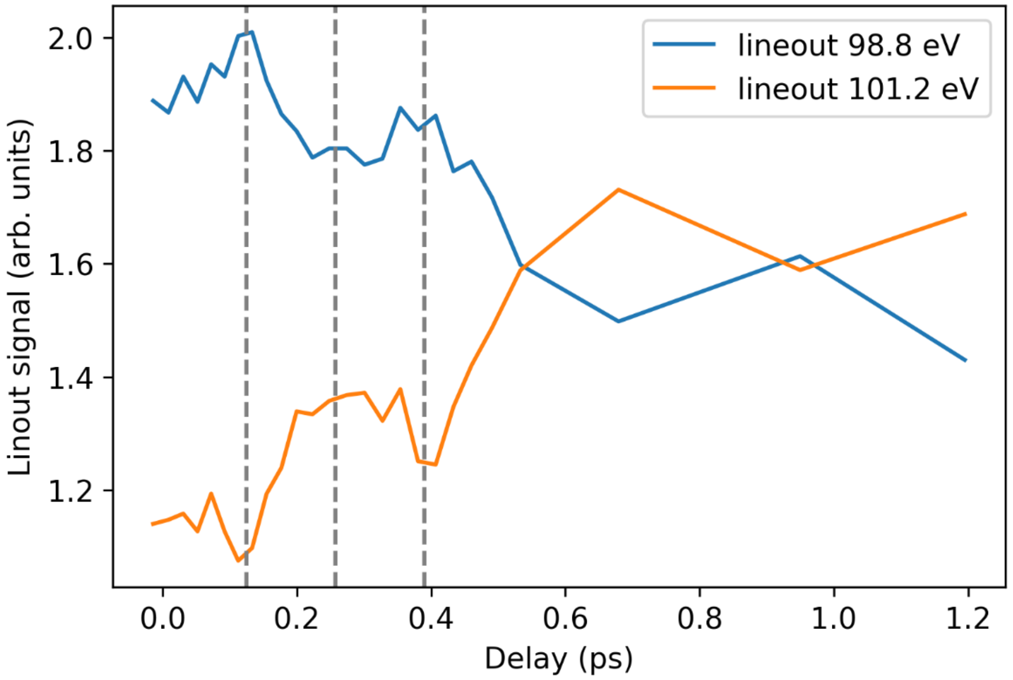

Supplementary Figure 5. Lineouts for the delay-dependent difference signal at 98.8 eV (blue line) and 101.2 eV (orange line). The two curves show out-of-phase oscillation within the first 600fs. The grey dashed vertical lines mark the same positions as the dashed lines in Fig. 3.

Supplementary Discussion 4: Quantum chemical results

The structure and the atom numbering of 2-thiouracil is shown in Suppl. Fig. 6. The most relevant molecular orbitals involved in the valence excitations are depicted in Suppl. Fig. 7 for the Franck-Condon geometry. The singlet states S_1 and S_2 have $n\pi^*$ and $\pi\pi^*$ character, respectively. At planar geometries, they originate from $27a'\rightarrow 7a''$ and $6a''\rightarrow 7a''$ transitions. The triplet states T_1 and T_2 originate mainly from the same transitions of S_2 and S_1, apart from the spin-flipping, and therefore they have $\pi\pi^*$ and $n\pi^*$ character, respectively. T_3 has a $\pi\pi^*$ character and is mostly contributed by the transitions $5a''\rightarrow 7a''$ and, to a lesser extent, $6a''\rightarrow 8a''$.

Table S2 reports the main structural parameters of the optimized geometries. The planar equilibrium structures are indicated by an asterisk ($S^*_{0,min}$, $S^*_{1,min}$, etc.); the non-planar geometry optimization of the states T_2 and T_3 did not converge to a stationary point. At all minima, except for $T^*_{1,min}$, the C–S bond is elongated by ≈ 0.1 Å as compared to $S_{0,min}$. A larger elongation of about 0.25 Å is found in the state S_2. In the ground state the difference between the planar and non-planar minima $S^*_{0,min}$ and $S_{0,min}$ is negligible. In contrast, the states S_1, S_2 and T_1 have non-planar minima which are depicted in Suppl. Fig. 8 and involve an out-of-plane distortion of the C–S bond, with pyramidalization angles in the range ≈ 35°–50°. The stabilization energy, associated with this out-plane distortion, is 0.30 eV, 0.48 eV and 0.17 eV for S_1, S_2 and T_1, respectively.

The comparison between Supplementary Table 2 and Table 2 of Ref. [2] shows that the present optimized gas phase geometries agree very well with those obtained using the multi-state CASPT2 method. The main notable difference is a somewhat higher value of the pyramidalization angle p_{10324}, around the N atom bridging the C=S and C=O groups. Supplementary Table 3 reports instead the non-planar and nearly planar minima obtained in Ref. [3] for 2-thiouracil in the presence of water solvent charges, which also agree nicely with the structures found in this work. This suggests that the same sets of geometries can be in principle visited by the wave packet both in the gas phase and in solution.

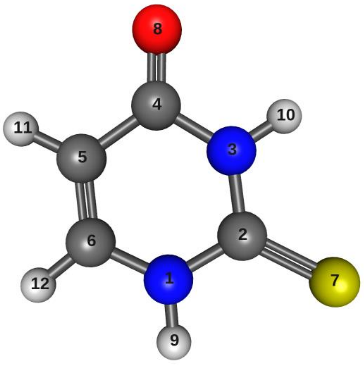

Supplementary Figure 6. Structure and atom numbering of 2-thiouracil. The carbons are gray, the nitrogens are blue, the hydrogens are white, the oxygen is red and the sulphur is yellow.

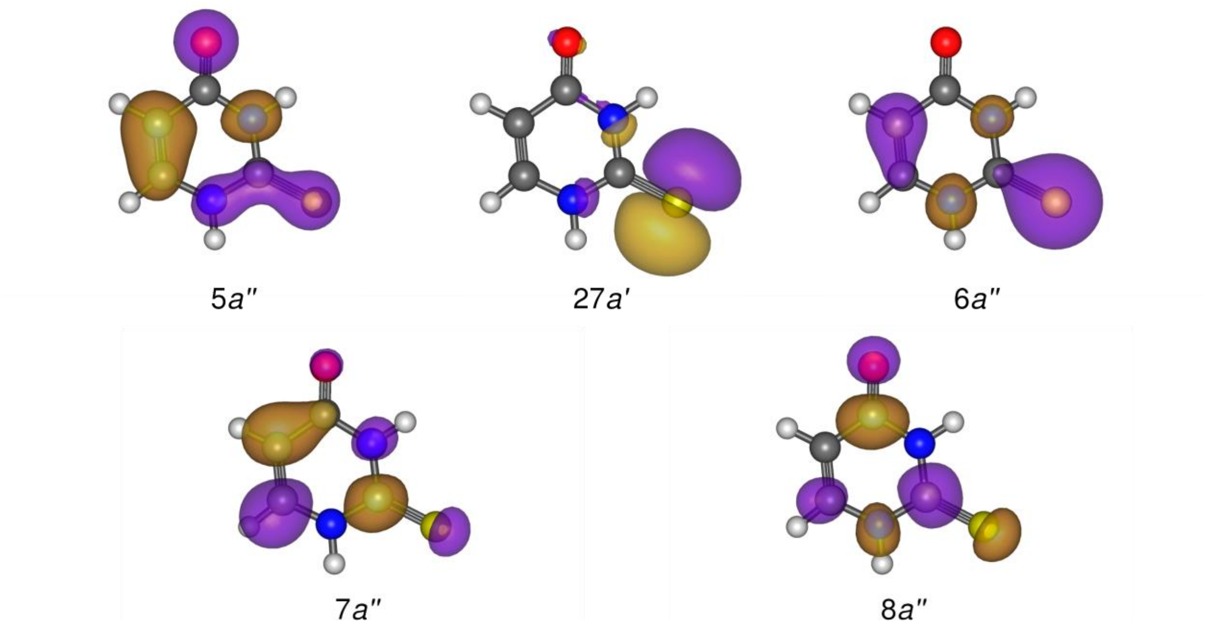

Supplementary Figure 7. Most relevant orbitals involved in the valence excitations of 2-thiouracil, evaluated at the $S^*_{0,min}$ geometry.

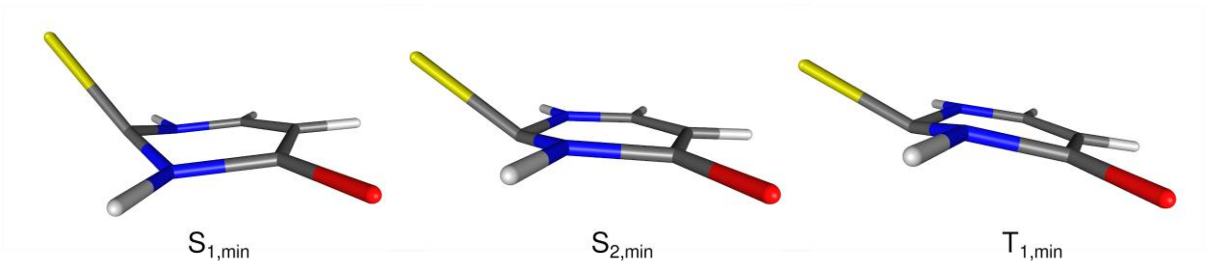

Supplementary Figure 8. Optimized structures of the non-planar minima of the excited states of 2-thiouracil.

	planar geometries					
	$S^*_{0,min}$	$S^*_{1,min}$	$S^*_{2,min}$	$T^*_{1,min}$	$T^*_{2,min}$	$T^*_{3,min}$
energy [eV]	0.00	3.78	4.44	3.17	3.65	3.76
r_{12}	1.37	1.39	1.32	1.36	1.40	1.41
r_{23}	1.37	1.40	1.35	1.39	1.41	1.37
r_{34}	1.41	1.40	**1.47**	1.41	1.39	1.41
r_{45}	1.47	1.47	**1.42**	1.44	1.47	1.44
r_{56}	1.35	1.36	1.41	1.47	1.36	1.41
r_{61}	1.38	1.37	1.39	1.36	1.37	1.39
r_{27}	1.65	**1.74**	**1.76**	1.67	**1.73**	**1.70**
r_{48}	1.21	1.22	1.22	1.22	1.22	1.23
α_{127}	122.8	122.1	120.5	121.7	122.2	121.3
α_{348}	120.4	120.3	**116.0**	120.2	120.3	119.7

	non-planar geometries			
	$S_{0,min}$	$S_{1,min}$	$S_{2,min}$	$T_{1,min}$
energy [eV]	0.00	3.48	3.96	3.00
r_{12}	1.37	1.40	1.37	1.40
r_{23}	1.37	1.40	1.38	1.41
r_{34}	1.41	1.40	1.41	1.40
r_{45}	1.47	1.47	1.47	1.47
r_{56}	1.35	1.35	1.35	1.36
r_{61}	1.38	1.38	1.38	1.37
r_{27}	1.65	**1.77**	**1.90**	**1.77**
r_{48}	1.21	1.21	1.21	1.21
α_{127}	122.8	**116.8**	**110.6**	**113.6**
α_{348}	120.4	121.0	120.9	121.2
p_{7213}	-1.5	**34.0**	**48.8**	**42.5**
p_{8435}	-0.6	-3.2	0.8	2.1
p_{10324}	8.0	19.8	-10.8	-22.1
p_{12651}	1.5	-2.5	0.5	0.7

Supplementary Table 2. Geometrical parameters of the planar and non-planar equilibrium structures of the lowest electronic states of 2-thiouracil, optimized at the (EOM-)CCSD/6-311++G** level. The largest structural changes with respect to $S_{0,min}$ are highlighted in boldface. The bond distances r_{ij} are given in Å, the valence angles α_{ijk} and the pyramidalization angles p_{ijkl} are in degrees. p_{ijkl} is defined as the angle between the vector of the *i–j* bond and the *jkl* plane. The dimension for the bond lengths is Å, for angles it is degrees.

	(nearly) planar geometries				
	$S^{*}_{0,min}$	$^1n\pi^*_{min}$	$^1\pi\pi^*_{min}$	$^3\pi\pi^*_{min}$	$^3n\pi^*_{min}$
r_{12}	1.36	1.34	1.33	1.35	1.32
r_{23}	1.37	1.37	1.34	1.39	1.35
r_{34}	1.38	1.44	**1.46**	1.41	1.45
r_{45}	1.43	1.40	**1.39**	1.41	1.39
r_{56}	1.36	1.41	1.41	1.44	1.41
r_{61}	1.36	1.37	1.41	1.35	1.40

r_{27}	1.65	**1.74**	**1.73**	1.69	**1.74**
r_{48}	1.25	1.25	1.27	1.25	1.25
α_{127}	123.9	122.3	120.8	123.1	121.2
α_{348}	120.5	116.2	**115.9**	117.2	115.9
p_{7213}	0.0	-1.2	-0.2	0.1	-1.6
p_{8435}	0.3	1.2	-0.2	1.3	1.1
p_{10324}	-9.0	**-19.1**	**-11.2**	**-10.7**	-11.2
p_{12651}	-0.3	-6.6	-7.8	-4.1	-3.5
	non-planar geometries				
	$S_{0,min}$	$^1n\pi^{*\prime}_{min}$	$^1\pi\pi^{*\prime}_{min}$	$^3\pi\pi^{*\prime}_{min}$	$^3n\pi^{*\prime}_{min}$
r_{12}	1.36	1.39	1.37	1.39	1.39
r_{23}	1.37	1.41	1.39	1.41	1.41
r_{34}	1.38	1.38	1.38	1.37	1.37
r_{45}	1.43	1.44	1.44	1.44	1.45
r_{56}	1.36	1.37	1.37	1.38	1.37
r_{61}	1.36	1.35	1.36	1.34	1.36
r_{27}	1.65	**1.79**	**1.86**	**1.77**	**1.78**
r_{48}	1.25	1.25	1.24	1.25	1.25
α_{127}	123.9	**118.4**	**112.6**	**115.8**	111.2
α_{348}	120.5	119.7	120.9	120.6	120.9
p_{7213}	-0.0	**32.2**	**45.6**	**37.5**	**41.5**
p_{8435}	0.3	-1.5	0.3	0.4	0.0
p_{10324}	-9.0	9.5	4.2	6.7	6.5
p_{12651}	-0.3	-0.1	0.6	0.2	-0.4

Supplementary Table 3. Geometrical parameters of the nearly planar and non-planar equilibrium structures of the lowest electronic states of 2-thiouracil, reported by Teles-Ferreira et al.[5]. The largest structural changes with respect to $S_{0,min}$ are highlighted in boldface. The bond distances r_{ij} are given in Å, the valence angles α_{ijk} and the pyramidalization angles p_{ijkl} are in degrees. p_{ijkl} is defined as the angle between the vector of the *i*–*j* bond and the *jkl* plane. The dimension for the bond lengths is Å, for angles it is degrees.

The EOM-CCSD electronic energies at the planar and non-planar minima of the different electronic states are reported in Supplementary Table 4 and plotted in Suppl. Fig. 9. The excitation energies at the ground state equilibrium ($S_{0,min}$) are larger by ≈ 0.4 eV compared to

those obtained by multi-reference calculations, although the spacing between the levels is similar. Indeed, the $S_2 \leftarrow S_0$ vertical excitation energy agrees quite well with the experimental gas phase UV absorption spectrum of thiouracil [4]. As shown in Suppl. Fig. 9, the potential energy surfaces of the states S_1 and T_2, both of *nπ** character, are nearly parallel.

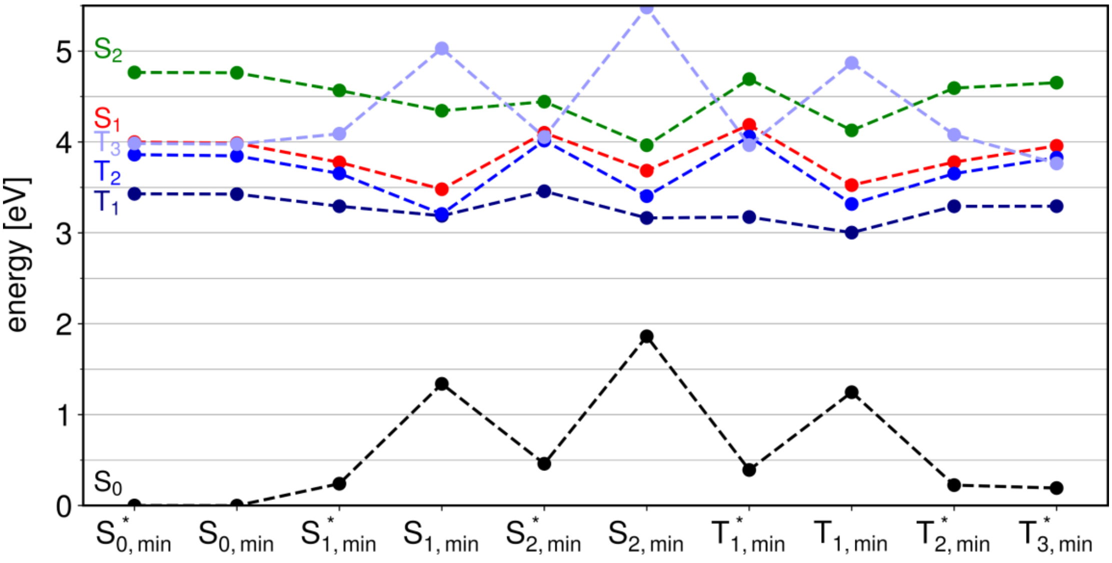

Supplementary Figure 9. EOM-CCSD/6-311++G** electronic energies of the lowest singlet and triplet states of 2-thiouracil, calculated at the planar and non-planar minima of the different states.

	State					
	S_0	S_1	S_2	T_1	T_2	T_3
	-	^{1}(27*a*'→7*a*'')	^{1}(6*a*''→7*a*'')	^{3}(6a''→7*a*'')	^{3}(27*a*'→7*a*'')	^{3}(5*a*''→7*a*'')<br>^{3}(6*a*''→8*a*'')
$S^*_{0,min}$	0.00	4.00	4.77	3.43	3.86	3.98
$S_{0,min}$	0.00	3.99	4.76	3.43	3.85	3.98
$S^*_{1,min}$	0.24	3.78	4.57	3.29	3.65	4.09
$S_{1,min}$	1.34	3.48	4.34	3.19	3.21	5.03
$S^*_{2,min}$	0.46	4.10	4.44	3.46	4.02	4.05
$S_{2,min}$	1.86	3.68	3.96	3.16	3.40	5.48
$T^*_{1,min}$	0.39	4.19	4.69	3.17	4.06	3.97
$T_{1,min}$	1.25	3.53	4.13	3.00	3.32	4.87

$T^*_{2,min}$	0.22	3.78	4.59	3.29	3.65	4.08
$T^*_{3,min}$	0.19	3.96	4.65	3.29	3.82	3.76

Supplementary Table 4. EOM-CCSD/6-311++G** electronic energies (in eV) of the lowest singlet and triplet states of 2-thiouracil, calculated at the planar and non-planar minima of the different states. For each state the dominant orbital transitions are reported. Energies are given in eV.

Supplementary Discussion 5: Calculated state- and geometry-dependent pump-probe XPS spectra

Photoelectron spectra are calculated at different geometries considering an ionisation process starting from the states S_1, S_2, T_1, T_2 and T_3. The reference "pump-off" spectrum, with ionisation starting from S_0, is calculated only at the ground state minimum. For each geometry *x* the spectrum $\sigma_n^x(E)$ from the state n is given as a sum of contributions associated with the three 2*p* orbitals,

$$\sigma_n^x(E) = C \sum_{i=1}^{3} A_{in}^x \, g(E - E_{in}^x),$$

where C is a constant, E_{in}^x is the ionization potential from the $2p_i$ orbital for the state *n* at the geometry *x*, evaluated by the EOM-IP-CCSD approach, and A_{in}^x is the ionization probability, approximated as the geometric average between the norms of the left and right Dyson orbitals[5]. The function *g*(·) is used to broaden the stick transitions in order to allow the comparison with experiment; the calculated profiles are obtained by applying a Gaussian broadening with a standard deviation of 1.5 eV. Since in the EOM-IP-CCSD procedure the valence excited states are described by unrestricted wavefunctions, the two binding energies for the α and β electrons differ by 0.0-0.1 eV and have been averaged in the calculation of E_{in}^x. The (geometry-dependent) pump-probe signal $S_n^x(E)$, associated with population on a given state, is obtained as the difference

$$S_n^x(E) = \sigma_n^x(E) - \sigma_{S_0}^{S_{0,min}}(E) .$$

The calculated pump-probe spectra are shown in Supplementary Figures 10 and 11 for the planar and non-planar geometries, respectively. Note the general trend for the binding energies $T_3 < S_2 < T_1 < S_1 \approx T_2$.

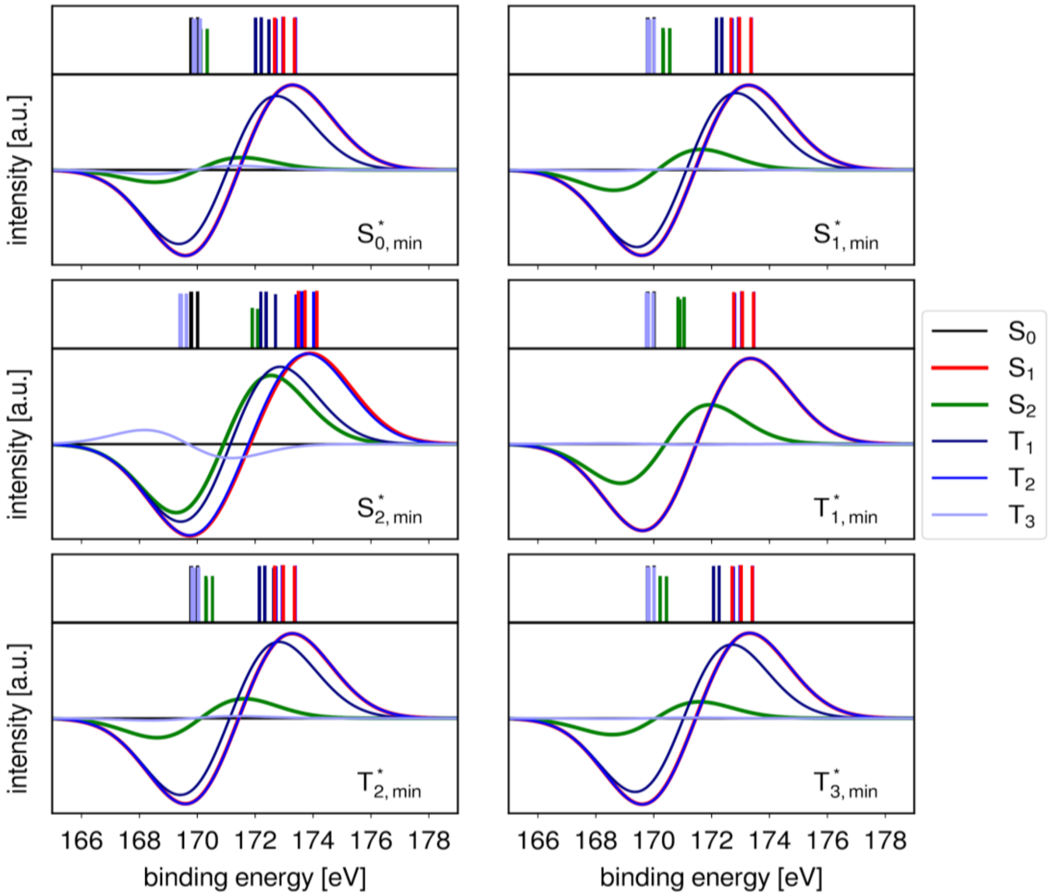

Supplementary Figure 10. Binding energies of the 2*p* electrons (top panels) and pump-probe spectra (bottom panels) evaluated for different electronic states and different planar stationary geometries. The ionization intensities are estimated using Dyson orbital norms.

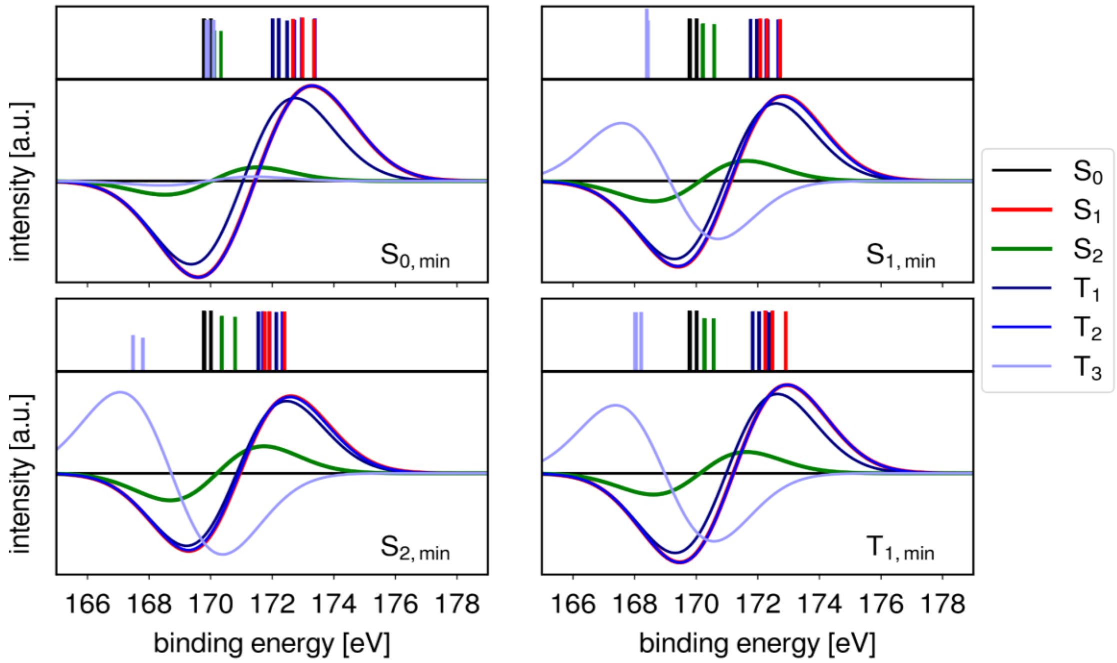

Supplementary Figure 11. Binding energies of the 2*p* electrons (top panels) and pump-probe spectra (bottom panels) evaluated for different electronic states and different fully optimized geometries. The ionization intensities are estimated using Dyson orbital norms.

Supplementary Discussion 6: Potential model fits

We extend the discussion of Figure 4b of the main text. Although the linear trend is already visible in Figure 4b, the relation between the local charge and binding energy (or excited state chemical shift) becomes even more obvious, when taking geometrical effects into account. In this, we closely follow the potential model as introduced by Gelius[6].

We fit two different versions of the potential model. Model A is a simple linear relation between the binding energy E_{bind} and local charge at the probed atom (sulphur) Q_S:

$$E_{\text{bind}} = k \cdot Q_S + l,$$

with *k* and *l* as fit constants.

We use the calculated Löwdin charges to perform a fit and deduce *k* and *l* from the dataset in Figure 4b. The values from this fit are plotted against the model potential binding energy on the x-axes and the ab initio binding energy on the y-axis in Supplementary Figure 12. This model leads to an R^2 of 0.82.

A better fit can be obtained when taking the charge and geometry of the environment of the sulphur atom into account:

$$E_{\text{bind}} = k \cdot Q_S + \sum_{A \neq S} Q_A / R_{AS} + l,$$

where the Q_S are the charges at all other atoms at their respective distances to the probed sulphur atom R_{AS}. The result of the fit is shown in Supplementary Figure 13. It makes the fit even better, increasing the R^2 from 0.82 in the simple linear potential model to 0.92 in the more sophisticated potential model.

The remaining discrepancies between the potential model and the ab initio binding energies are due to final state effects including electronic relaxation. These effects have been discussed in the static literature (see for example Ref. [7]).

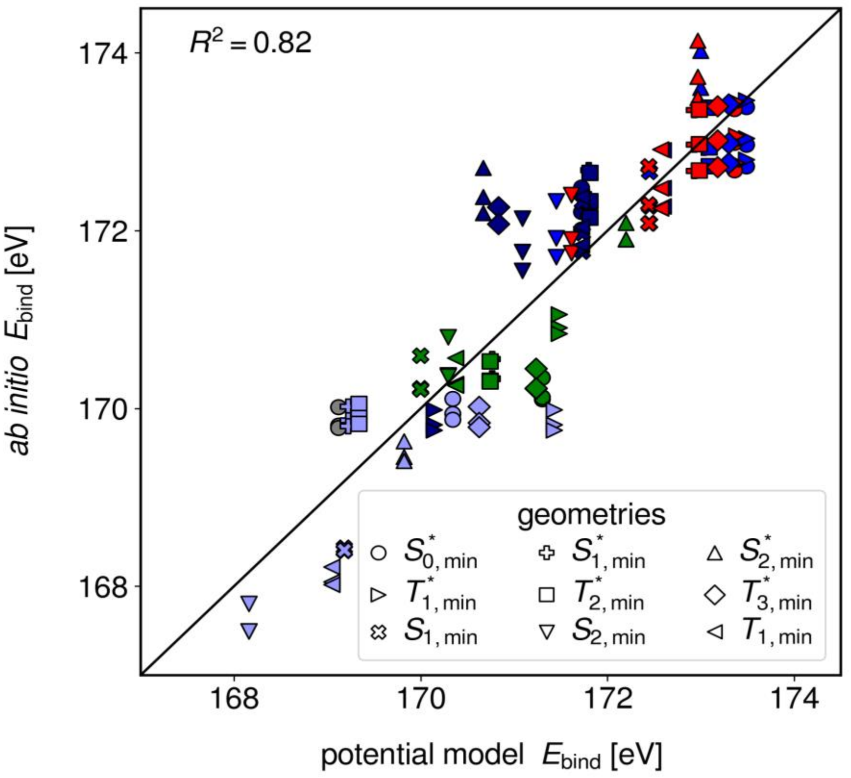

Supplementary Figure 12. Binding energy from the ab initio calculations vs binding energy from the simple potential model for the dataset in Figure 4b.

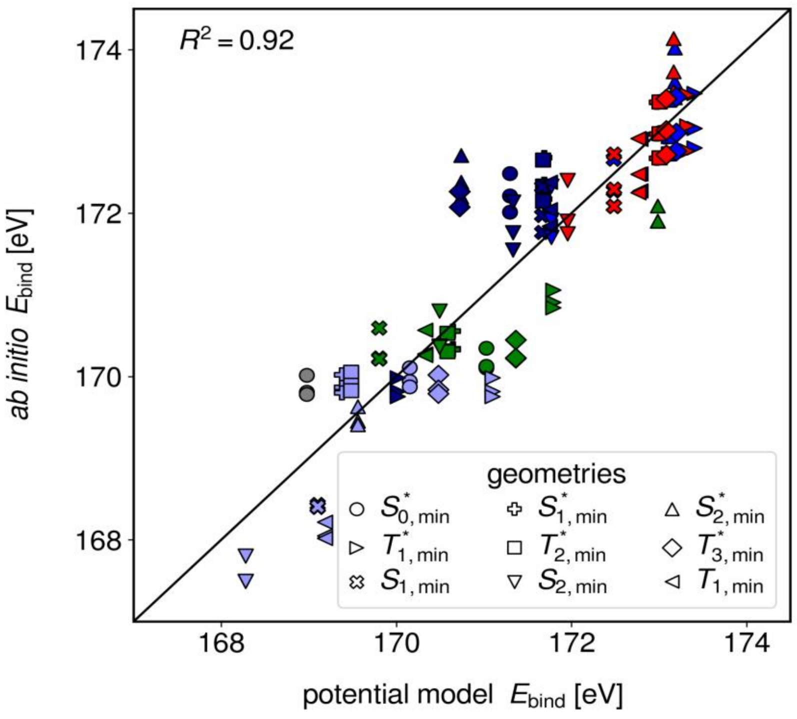

Supplementary Figure 13. Same as Supplementary Figure 12 but using a geometrically corrected potential model.

Supplementary Discussion 7: Exceptions in the clustering according to electronic state

The data of Suppl. Table 2 allows us to explain the exceptions in the clustering according to electronic states reported in Figure 4(b) of the main text. For example, the dots for the T_1 state at the planar T_1 minimum ($T^*_{1,min}$) are quite separated from the rest of the T_1 cluster. A probable reason is that this geometry is quite similar to the Frank-Condon (FC) point, except for a slight elongation of the C=C bond. In particular, in contrast to the other excited state minima, the C-S bond distance is relatively short. Therefore, it is easier for the electron hole created by UV excitation on the S atom to redistribute partially on the neighbouring atoms. Then, the 2*p* binding energy decreases.

The opposite is true for the dots of the S_2 state at the planar S_2 minimum ($S^*_{2,min}$). Indeed, among the planar geometries, this is the one with the largest C-S bond distance, so that the hole is more likely to localize on the S atom.

Note that, although these points shift away from their clusters, the shift always correlates with the electron binding energy: the higher the partial charge on the S atom, the higher the ionization potential.

Supplementary Discussion 8: Pulse energy scans

Power-dependent scans were performed for x-ray only probing (Suppl. Fig. 14) to assure that the x-ray induced photoelectron signal is not saturated. The resulting x-ray pulse energy distribution used in the experiment is shown in Suppl. Fig. 14c. Similarly, UV-power scans were performed (Suppl. Fig. 14) to avoid UV induced saturation effects. Here, the absolute of the total difference intensity is plotted as a function of UV pulse energy. The resulting pulse energy histogram with energies in the linear excitation regime is given in Suppl. Fig. 14d.

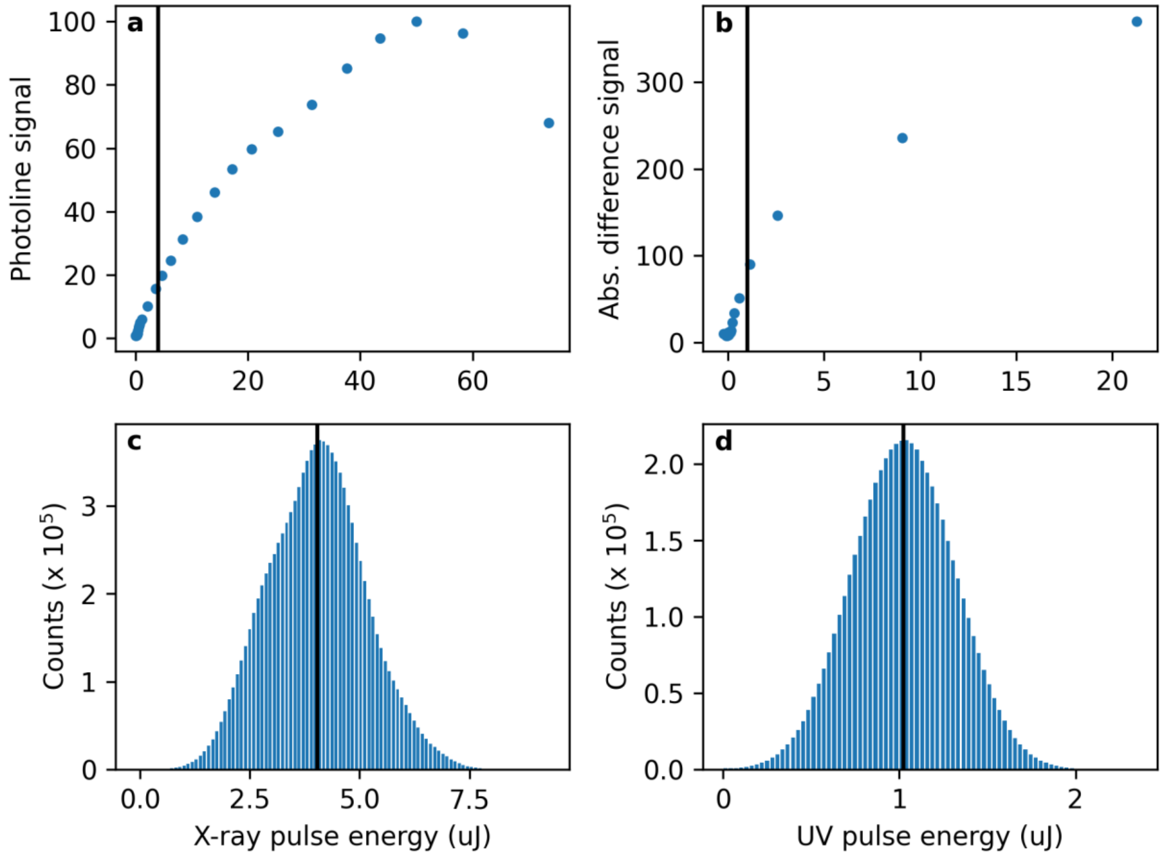

Supplementary Figure 14. Pulse energy scans for UV pump and x-ray probe pulse. a: X-ray-only S 2*p* photoelectron signal as a function of x-ray pulse energy. b: Integrated absolute difference signal at S 2*p* photoline as a function of UV pulse energy. c and d: X-ray and UV pulse energy distribution for the experimental data shown in the paper. The vertical black lines mark the centre of gravity of the distributions. They are also inserted in a & b for comparison to the pulse energy scans.

Supplementary Discussion 9: Data handling

Data handling procedure. The data presented covers about 8 million FEL shots. All electron time-of-flight traces have been normalised on FEL pulse energy prior to further processing. As shot-to-shot FEL photon energies are not delivered by FLASH, long term drifts and trends over the FEL pulse train have been tracked with the sulphur 2*p* photoline itself and corrected via self-referencing the photoline using the following procedure.

In order to determine the photoline position in the time-of-flight spectra better, a number of 200 consecutive pulse trains (20s of data, 50 pulses per train) were averaged pulse-wise. The resulting 50 spectra were fitted with a Gaussian function within a 200 ns window around the expected photoline position. However, only every second shot can be used to evaluate the influence of the FEL on the spectrum as the other half was additionally influenced by the UV pump. The trend over pulse train of the remaining 25 values is fitted with a second order polynomial. The resulting curve is used to shift the original 10,000 raw spectra. To track not only the trends over the pulse trains but also long-term shifts, the overall mean of the photoline position of the first of those processed data chunks is set as a global reference. The raw spectra for all data chunks are shifted towards this reference utilizing the (for each chunk determined) pulse train trend of the average spectra.

After that, shot-to-shot difference spectra were calculated and the resulting spectra were binned by delay. The delays were corrected by means of the bunch arrival monitor (BAM) which measures the arrival of the electron bunches and thus gives information on the arrival time jitter of the x-ray pulses[8,9]. The delay binning was chosen in such a way that all bins have similar statistics (~73,000 shot pairs per bin). In combination with the delay correction via BAM, this allows a finer binning for delays close to time-zero than experimentally set.

Influence of the data handling on the experimental spectra. Both the spectral fluctuations of the x-ray pulse and the pump-probe delay fluctuations are potentially smearing out spectral-temporal signatures of the molecule. These fluctuations are resulting from the fact that the free-electron-laser is starting its lasing process from noise, part of the temporal jitter is due to other stabilization issues. Several papers have been devoted to investigate the issues of delay jitter (in Suppl. Refs. [8,9]) and we systematically investigated the effect of spectral and timing jitter on time-resolved photoelectron spectra in Suppl. Ref. [10].

The delay time jitter in our data is about 300 fs. While the photon energy jitter primarily changes the position and the width of the photoline, this delay jitter may also change the shape of the observed photoline. Applying a correction to the delays by tracing the arrival time of the x-ray pulses already eliminates a significant part of the random fluctuations in the time-dependent difference spectra. This has been demonstrated before in Suppl. Refs. [8,9]. For the difference spectra here, it makes the spectral oscillations in the region between 100 and 101 eV become visible (see Figure 4b in Suppl. Ref [10]).

The most obvious 'missing' spectral feature is the sulphur photoelectron line spin-orbit splitting of 1.2 eV, that cannot be identified in our data. Since we do not have a single-shot spectral tool available, we used self-referencing for spectral correction. Based on a combination of simulations and data, we found that shot-to-shot correction by self-referencing of the photoline can correct the jitter and drifts in the static case. For pump-probe difference spectra, however, the correction of unpumped shots can only be achieved by well-adapted averaging of the data and utilising correlations in the pulse train of the FEL. Nonetheless, this allows to correct long-term drifts of the FEL photon energy. Thus, our method does not influence the shot-to-shot statistics, but narrows the long-term averaged statistics and improves it by a factor of 2-3.

Supplementary References

1. Kruit, P. & Read, F. H. Magnetic field paralleliser for 2π electron-spectrometer and electron-image magnifier. *J. Phys. E.* **16**, 313–324 (1983).
2. Mai, S., Marquetand, P. & González, L. A Static Picture of the Relaxation and Intersystem Crossing Mechanisms of Photoexcited 2-Thiouracil. *J. Phys. Chem. A* **119**, 9524–9533 (2015).
3. Teles-Ferreira, D. C. *et al.* A Unified Experimental/Theoretical Description of the Ultrafast Photophysics of Single and Double Thionated Uracils. *Chem. - A Eur. J.* **26**, 336–343 (2020).
4. Khvorostov, A., Lapinski, L., Rostkowska, H. & Nowak, M. J. UV-induced generation of rare tautomers of 2-thiouracils: A matrix isolation study. *J. Phys. Chem. A* **109**, 7700–7707 (2005).
5. Vidal, M. L., Krylov, A. I. & Coriani, S. Dyson orbitals within the fc-CVS-EOM-CCSD framework: Theory and application to X-ray photoelectron spectroscopy of ground and excited states. *Phys. Chem. Chem. Phys.* **22**, 2693–2703 (2020).
6. Gelius, U. Binding Energies and Chemical Shifts in ESCA. *Phys. Scr.* **9**, 133–147 (1974).
7. Mårtensson, N. & Nilsson, A. On the origin of core-level binding energy shifts. *J. Electr. Spectr. Rel. Phen.* **75**, 209–223 (1995).
8. Schulz, S. *et al.* Femtosecond all-optical synchronization of an X-ray free-electron laser. *Nat. Commun.* **6**, 5938 (2015).
9. Savelyev, E. *et al.* Jitter-correction for IR/UV-XUV pump-probe experiments at the FLASH free-electron laser. *New J. Phys.* **19**, 43009 (2017).
10. Mayer, D., Lever, F. & Gühr, M. Data analysis procedures for time-resolved x-ray photoelectron spectroscopy at a SASE free-electron-laser. *J. Phys. B At. Mol. Opt. Phys.* (2021) doi:10.1088/1361-6455/ac3c91.
